# The Impact of Cr^3+^ Doping on Temperature Sensitivity Modulation in Cr^3+^ Doped and Cr^3+^, Nd^3+^ Co-doped Y_3_Al_5_O_12_, Y_3_Al_2_Ga_3_O_12_, and Y_3_Ga_5_O_12_ Nanothermometers

**DOI:** 10.3389/fchem.2018.00424

**Published:** 2018-09-19

**Authors:** Karolina Elzbieciak, Lukasz Marciniak

**Affiliations:** Institute of Low Temperatures and Structure Research PAS, Wrocław, Poland

**Keywords:** chromium, luminescence, luminescent thermometers, Nanocrystals (NCs), garnets

## Abstract

A new approach to enhance the sensitivity of transition metal ion based nanocrystalline luminescent thermometer is presented. It was shown that the increase of Cr^3+^ concentration in three types of garnet host namely Y_3_Al_5_O_12_, Y_3_Ga_5_O_12_, and Y_3_Al_2_Ga_3_O_12_ allows for significant enhancement of their performance in non-contact thermometry. This phenomenon is related to the weakening of the crystal field strength due to enlargement of average Cr^3+^-O^2−^ distance at higher Cr^3+^ concentrations. By increasing Cr^3+^ concentration from 0.6 to 30%, the sensitivity increased by over one order of magnitude from *S* = 0.2%/°C to *S* = 2.2%/°C at 9°C in Y_3_Al_2_Ga_3_O_12_ nanocrystals. Moreover, it was found that due to the Cr^3+^ → Nd^3+^ energy transfer in the Cr^3+^, Nd^3+^ co-doped system, the usable Cr^3+^ concentration, for which its emission can be detected, is limited to 10% while the sensitivity at −50°C was doubled (from 1.3%/°C for Y_3_Al_2_Ga_3_O_12_:10%Cr^3+^ to 2.2%/°C Y_3_Al_2_Ga_3_O_12_:10%Cr^3+^, 1%Nd^3+^ nanocrystals).

## Introduction

In response to the requirements imposed by technology, micro/nanoelectronics or photonics as well as by biomedical applications, new approaches to the luminescent nanothermometers (LNTs) have to be proposed to secure fast and accurate temperature sensing with submicrometer spatial resolution, and highly sensitive temperature readout (Brites et al., 2012; Jaque and Vetrone, 2012; Chen et al., 2016; del Rosal et al., 2016a,b; Dramićanin, 2016; Marciniak et al., 2016b; Suo et al., 2017; Wang et al., 2017; Gao et al., 2018; Liao et al., 2018; Liu et al., 2018; Malysa et al., 2018; Runowski et al., 2018; Zhong et al., 2018). One of the most promising one, relies on exploiting transition metal (TM) ions, whose highly temperature dependent emission is referred to emission of barely temperature dependent lanthanides ions (Marciniak et al., 2017a; Drabik et al., 2018; Elzbieciak et al., 2018; Kniec and Marciniak, 2018; Marciniak and Trejgis, 2018; Trejgis and Marciniak, 2018). Materials which could be applied as real time temperature sensors in biomedicine, must also accomplish some other important requirements like sufficient sensitivity to temperature changes, high stability, low cytotoxicity (Brites et al., 2012; Jaque and Vetrone, 2012; Benayas et al., 2015) and operation in spectral range of optical transparency windows of biological tissues (Anderson and Parrish, 1981; Jaque and Jacinto, 2016). Because the transition metal ions based luminescent nanothermometers meet abovementioned flagship demands, they can be considered as distinctively attractive research area. However, in order to be applicable, the thorough understanding of the correlation between structure of the host material and the thermal quenching of luminescence has to be studied. In our previous work (Elzbieciak et al., 2018), we have shown that by appropriate adjustment of the stoichiometry of the host matrix, relative sensitivity of Cr^3+^ ions-based luminescent thermometer can be intentionally modulated. The presented tuning occurred as a result of modification of the metal-to-oxygen ionic distance, which modified the strength of the crystal field (CF) (Struve and Huber, 2000; Xu et al., 2017). Therefore, taking advantage from the fact that CF influences the position of ^4^T_2_ parabola, the activation energy, responsible for determination of thermal stability of luminescence, can be deliberately reduced. Efficient thermal quenching of the luminescence intensity is beneficial for sensitive luminescent thermometry. As it was already presented (Marciniak and Bednarkiewicz, 2016; Marciniak et al., 2016a, 2017b; Azkargorta et al., 2017), significant changes of the CF strength may also result from rising the concentration of active or passive dopants. Recently, Deren et al. (2012) showed that the increase of the Cr^3+^ concentration in Y_3_Ga_5_O_12_:Cr^3+^ causes the diminishment of ^2^E → ^4^A_2_ narrow emission band and enhancement of ^4^T_2_→^4^A_2_ band's intensity. Lowering the CF strength by growing number of Cr^3+^ ions, which is related with the elongation of the M-O distance, should relevantly enhance the relative sensitivity to temperature changes of such Cr^3+^ based luminescent thermometer. These observations motivated us to verify this hypothesis through comprehensive investigations of the impact of Cr^3+^ concentration (0.01–50%) on the temperature sensing capability in Y_3_Al_5_O_12_, Y_3_Al_2_Ga_3_O_12_ and Y_3_Ga_5_O_12_ nanocrystals.

## Experimental

Nanopowders of (0.1; 0.5; 2; 5; 10; 20; 50%) Cr^3+^ and (0.1; 0.5; 2; 5; 10; 20; 50%) Cr^3+^,1% Nd^3+^ doped Y_3_Al_5_O_12_, Y_3_Ga_5_O_12_ and (0.06; 0.3; 1.2; 3; 6; 9; 12; 30%) Cr^3+^, (0.06; 0.3; 1.2; 3; 6; 9; 12; 30%) Cr^3+^, 1%Nd^3+^ doped Y_3_Al_2_Ga_3_O_12_ garnets were synthesized by the modified Pechini method (Pechini, 1967). Whole synthesis procedure was analogous as described in our earlier work (Elzbieciak et al., 2018). In brief, in order to obtain metal nitrates, calculated amount of yttrium oxide and neodymium oxide were dissolved in deionized water with addition of ultrapure nitric acid. After triple recrystallization process, yttrium or yttrium and neodymium nitrates were mixed together with aluminum, gallium and chromium nitrates. Afterwards, aqueous solution of citric acid and PEG was added to the mixture, than it was stirred for 3 h. In order to form a resin the obtained solution was heated for 1 week at 90°C.

All the synthesized materials were annealed for 16 h at 850°C. The following starting materials were used for synthesis: yttrium oxide (Y_2_O_3_ with 99.995% purity from Stanford Materials Corporation), neodymium oxide (Nd_2_O_3_ with 99.998% purity from Stanford Materials Corporation), aluminum nitrate nonahydrate (Al(NO_3_)_3_·9H_2_O Puratronic 99.999% purity from Alfa Aesar), gallium(III) nitrate nonahydrate (Ga(NO_3_)_3_·9H_2_O Puratronic 99.999% purity from Alfa Aesar), chromium nitrate nonahydrate (Cr(NO_3_)_3_·9H_2_O, 99.99% purity from Alfa Aesar), citric acid (C_6_H_8_O_7_ with 99.5+% purity from Alfa Aesar) and poly(ethylene glycol) (PEG C_2_H_6_O_2_ BioUltra 200 from Sigma).

All of the obtained materials were examined by XRD (X-ray diffraction) measurements carried out on PANalitycal X'Pert diffractometer, equipped with an Anton Paar TCU 1000 N temperature control unit, using Ni-filtered Cu-K_α_ radiation (*V* = 40 kV, *I* = 30 mA). Transmission electron microscope (TEM) images were taken using FEI TECNAI G2 X-TWIN microscope equipped with EDS detector. Powders were dispersed in methanol solution in ultrasounds and applied for lacey type copper lattices. The studies were performed in conventional TEM microscope with 300 keV parallel beam electron energy. Images were digitally recorded using the Gatan Ultrascan 1000XP.

Excitation spectra were measured using FLS980 Fluorescence Spectrometer form Edinburgh Instruments. Temperature dependent emission spectra were measured using 450 nm excitation line from laser diode and recorded using a Silver-Nova Super Range TEC Spectrometer from Stellarnet of 1 nm spectral resolution. Temperature during the measurement was controlled using the THMS 600 heating stage from Linkam (0.1°C stability and 0.1°C set point resolution).

## Results and discussion

**Figure 1:**
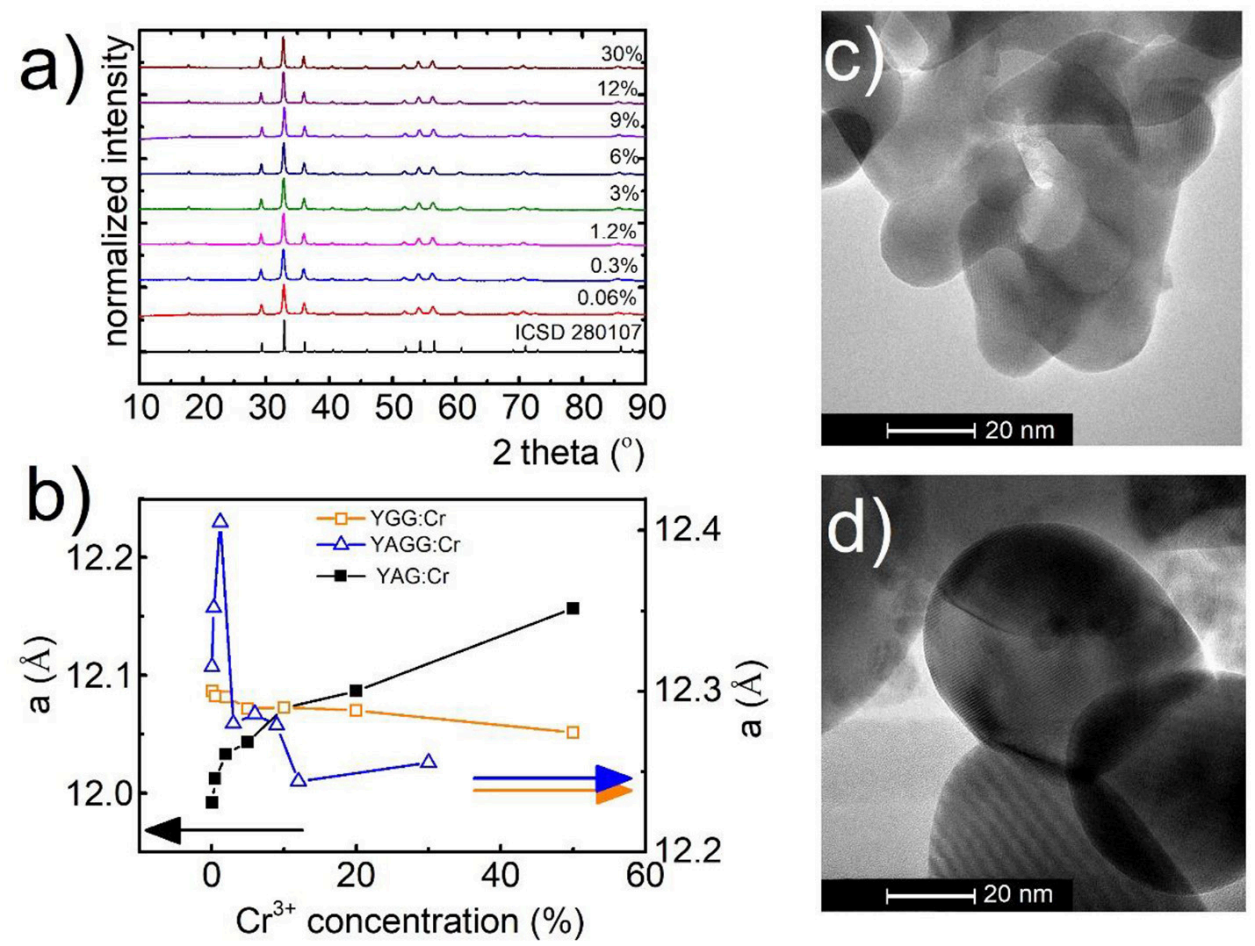
Structural and morphological characterization of synthesized materials: XRD spectra for Y_3_Al_2_Ga_3_O_12_ **(a)** with different concentrations of Cr^3+^ ions, dependence of unit cell parameter **(a)** of YGG, YAG, and YAGG on Cr^3+^ concentration **(b)**; representative TEM images for Y_3_Al_2_Ga_3_O_12_:0.06%Cr^3+^,1%Nd^3+^ **(c)** and Y_3_Al_2_Ga_3_O_12_:30%Cr^3+^,1%Nd^3+^**(d)** nanocrystals.

The yttrium aluminum/gallium garnets, crystallize in cubic structure with Ia3-d space group. As it is known, structures of these materials (A_3_B_2_C_3_O_12_) provide three types of cation sites, namely dodecahedral Y^3+^ site and also octahedral (B) and tetrahedral (C) Al^3+^/Ga^3+^ sites which, because of similarity in ionic radii and the same coordination number, could be occupied by lanthanide Ln^3+^ (A) and transition metal ions TM^3+^ (B, C), respectively. Representative XRD patterns of Y_3_Al_2_Ga_3_O_12_: Cr^3+^ nanocrystals with different chromium concentration are presented in Figure 1a. All the reflection peaks correspond to the reference patterns confirming phase purity of the synthesized materials even for high dopant concentration (see also Supporting Information, Figures S1–S5). It is worth noting that in case of YAG (Figures S2, S3) and YGG (Figures S4, S5) with doping above 20% of Cr^3+^ ions, additional peaks occur in the XRD pattern. The cell parameter, as it can be seen in Figure 1b, is strongly affected by the Cr^3+^ concentration. In the case of YAG:Cr^3+^, parameter *a* increases from 11.99 Å for 0.1%Cr^3+^ to 12.16 Å for 50%Cr^3+^. On the other hand for YGG:Cr^3+^ an opposite tendency can be found –the *a* parameter decreases from 12.09 Å for 0.1%Cr^3+^ to 12.05 Å for 50%Cr^3+^. This is due to differences in ionic radius between host ions Al^3+^ (67.5 pm) and Ga^3+^(76 pm) ions being substituted by a larger dopant Cr^3+^ ions (75.5 pm). When atom with shorter ionic radius is substituted by atom with longer one, as in the case of Y_3_Al_5_O_12_, the volume of the unit cell increases, due to the local expansion of the structure. This was also confirmed by the increase of the microstrains in the YAG structure (calculated using Rietveld refinement, Figure S7). On the other hand, in the case of substitution of larger host ion with a smaller dopant ion, the parameter *a* and microstrains decrease likewise in the Y_3_Ga_5_O_12_. The reduction of the cell parameter for increasing Cr^3+^ concentration observed in YAGG, is therefore a direct confirmation that Cr^3+^ ions substitute octahedral sites of Ga^3+^ ions. The average grain size of the nanocrystals, calculated using Rietveld refinement technique (around 60 nm), was in agreement with the nanoparticle size distribution determined from TEM images-70 ± 10 nm (Figures 1c,d, Figure S6). TEM images revealed good crystallization and some agglomeration of the obtained powders.

**Figure 2:**
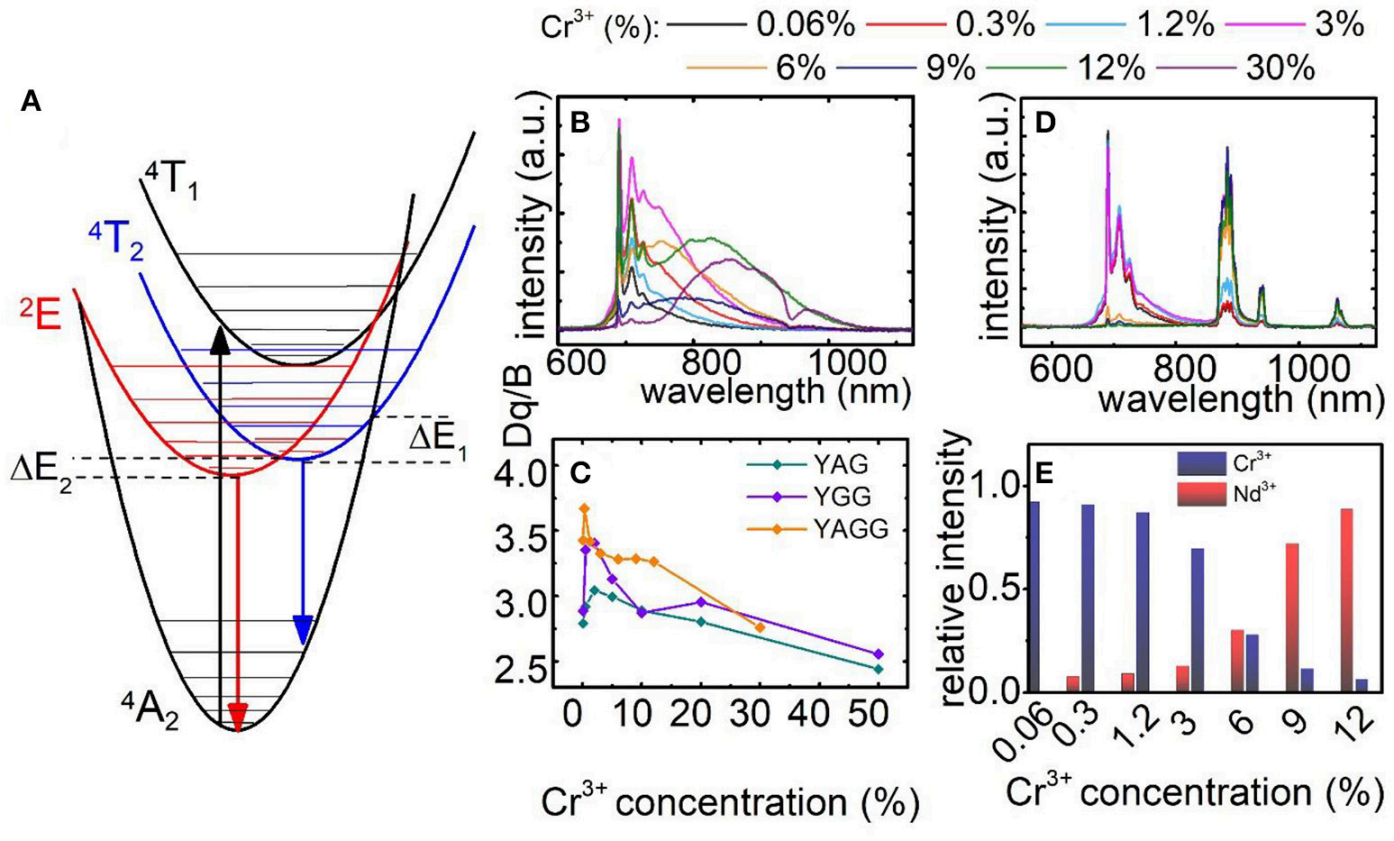
Configurational coordinates diagram of Cr^3+^ ions **(A)**; comparison of normalized emission spectra for Y_3_Al_2_Ga_3_O_12_: xCr^3+^ **(B)** and for Y_3_Al_2_Ga_3_O_12_: xCr^3+^, 1%Nd^3+^ nanocrystals **(D)**; dependence of Dq/B parameter **(C)** and Cr^3+^/Nd^3+^ ratio at *T* = −150°C **(E)** on Cr^3+^ concentration.

The simplified configurational coordinates diagram of Cr^3+^ is presented in Figure 2A. The luminescence of Cr^3+^ ions occurs through radiative depopulation of ^2^E and/or ^4^T_2_ states to the ^4^A_2_ ground state. Due to the fact that strength of the crystal field determines the emission of Cr^3+^ ions, sharp emission line corresponding to the ^2^E → ^4^A_2_ transition and broadband emission corresponding to the ^4^T_2_→^4^A_2_ transition can be observed in the emission spectra of Cr^3+^ ion in strong and weak crystal field, respectively. At higher temperatures, when the thermal energy is sufficient to reach the intersection point between the ^2^E parabola and ^4^T_2_ or ^4^A_2_ parabolas, the process of nonradiative, multiphonon relaxation results in lowering of their emission intensity. Analysis of the emission spectra of Y_3_Al_2_Ga_3_O_12_:Cr^3+^ for different dopant concentration obtained upon 450 nm excitation, clearly indicates that at higher Cr^3+^ concentration the broad ^4^T_2_→^4^A_2_ emission band localized at around 870 nm increase its intensity in respect to the ^2^E → ^4^A_2_ band at 692 nm (Figure 2B). Obviously, the total emission intensity decreases at higher dopant concentration due to the concentration quenching of luminescence. Nevertheless, it can be distinctly seen that ^4^T_2_→^4^A_2_ dominates in the spectra for 30% of Cr^3+^ ions. Similar observation can be done in the emission spectra of Y_3_Al_5_O_12_:Cr^3+^ and Y_3_Ga_5_O_12_:Cr^3+^ nanocrystals presented in Figures S8, S9, respectively. However, in these cases similar trends can be observed for lower Cr^3+^ concentrations and to a lesser extent also in Y_3_Al_2_Ga_3_O_12_. Two main consequences of the lowering of ^4^T_2_ state parabola can be found. First, at low dopant concentration the gradual reduction of its energy facilitates thermal depopulation of ^2^E state–lowered ΔE_2_ energy (the consequence of the intersection point between ^2^E and ^4^T_2_ parabolas). Secondly, at higher dopant concentration, when the energy ^4^T_2_ state becomes lower than ^2^E one, the broadband emission appears. The characteristic red-shift of the Cr^3+^ absorption bands localized around 400 nm and 575 nm, which can be attributed to the ^4^A_2_→^4^T_1_ and ^4^A_2_→^4^T_2_ electronic transitions, respectively, is a result of lowering of the CF strength (Figures S12–S14). In order to quantify the observed change of the crystal field strength in the examined nanocrystals, the Dq/B parameter was calculated for each of the samples as follows (Casalboni et al., 1994):

(1) $$Dq=\frac{E\left({}^{4}A_{2}\to^{4}T_{2}\right)}{10}$$

(2) $$\begin{array}{r} \frac{Dq}{B}=\frac{15\left(x-8\right)}{\left(x^{2}-10x\right)} \end{array}$$

where x could be defined as (Casalboni et al., 1994):

(3) $$x=\frac{E\left({}^{4}A_{2}\to^{4}T_{1}\right)-E\left({}^{4}A_{2}\to^{4}T_{2}\right)}{Dq}$$

As it can be observed in Figure 2C the Dq/B gradually decreases with Cr^3+^ concentration from 2.79 to 2.44 for Y_3_Al_5_O_12_; from 2.88 to 2.55 for Y_3_Ga_5_O_12_ and from 3.42 to 2.76 for Y_3_Al_2_Ga_3_O_12_ nanocrystals. In order to obtain self-referenced luminescent thermometer based of the Cr^3+^ emission intensity the examined nanocrystals with different Cr^3+^ concentration were co-doped with 1% Nd^3+^ ions. Emission intensity of Nd^3+^ is expected to be significantly less temperature dependent in respect to the chromium emission. Moreover, the Nd^3+^ ions emit in the first (band around 880 nm attributed to the ^4^F_3/2_→^4^I_9/2_ electronic transition) and the second (bands around 1064 nm and 1350 nm attributed to the ^4^F_3/2_→^4^I_11/2_ and ^4^F_3/2_→^4^I_13/2_ electronic transitions, respectively) optical window of the biological tissues making it well suited for biological applications.

Following excitation spectra, a 450 nm excitation line was chosen, which provides the condition of direct excitation of each individual optically active ion-Cr^3+^ (^4^A_2_→^4^T_1_) and independently Nd^3+^ (^4^I_9/2_→^2^G_5/2_). As it was recently showed, this is an important condition to enhance the relative sensitivity of this kind of LTs (Marciniak et al., 2017a). In case of Y_3_Al_2_Ga_3_O_12_: Cr^3+^, Nd^3+^ (Figure 2D) nanocrystals the presence of Nd^3+^ ion significantly quenched the Cr^3+^ emission intensity due to the Cr^3+^ → Nd^3+^ energy transfer. Therefore, the ^2^E → ^4^A_2_ emission band can be barely seen for Y_3_Al_2_Ga_3_O_12_: 30%Cr^3+^, 1%Nd^3+^ while in the case of Y_3_Al_5_O_12_ and Y_3_Ga_5_O_12_ above 10% of Cr^3+^, no chromium emission was detected (Figures S10, S11). Moreover, the presence of broad Cr^3+^ absorption bands in the excitation spectra when monitoring Nd^3+^ emission (^4^F_3/2_→^4^I_9/2_ emission band), is an additional confirmation of the interionic energy transfer which takes place between dopants (Figures S12–S14). The change of contribution of the emission of particular optically active ions in the total emission intensity related with Cr^3+^ concentration is presented in Figure 2E. Initially, at low Cr^3+^ amount the ^2^E → ^4^A_2_ emission band dominates in the spectra. However, around 6% of Cr^3+^ its emission intensity equalize with Nd^3+^ amount. Above this value, the chromium emission intensity rapidly decreases. Therefore, this energy transfer strongly limits the usable Cr^3+^ concentration which can be applied for luminescent thermometry.

**Figure 3:**
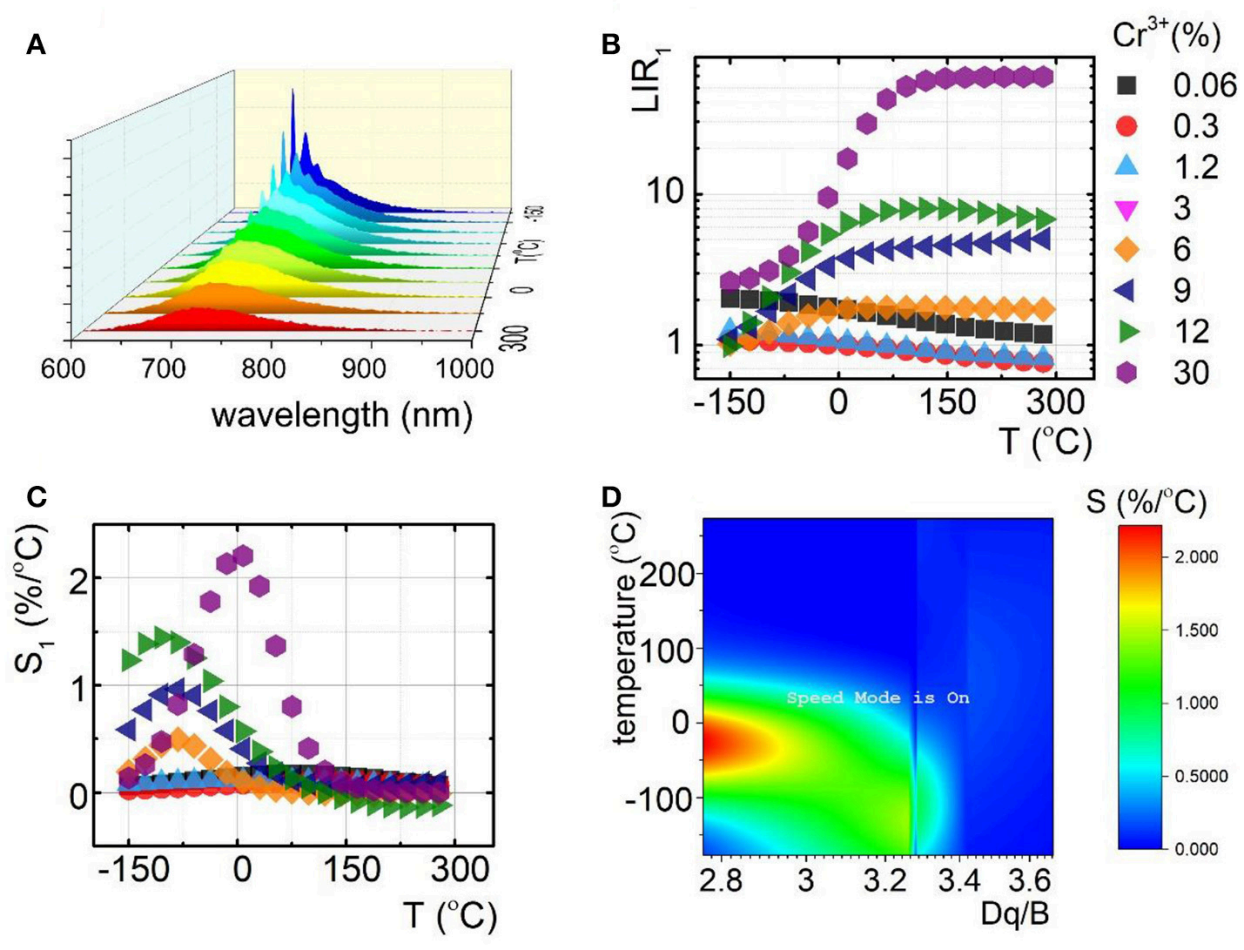
Comparison of thermal evolution of emission spectra for Y_3_Al_2_Ga_3_O_12_:1.2%Cr^3+^ **(A)**; evolution of LIR_1_ **(B)** with the corresponding relative sensitivities (S_1_) **(C)** in the function of temperature; sensitivity map for different temperatures depending on Dq/B **(D)**.

To understand the role of Cr^3+^ on the luminescence thermal quenching in the Y_3_Al_5_O_12_ and Y_3_Ga_5_O_12_ and Y_3_Al_2_Ga_3_O_12_ nanocrystals, their emission spectra were measured in a wide temperature range. The representative thermal evolution spectrum of Y_3_Al_2_Ga_3_O_12_:1.2%Cr^3+^ nanocrystals is presented in Figure 3A. It is clearly seen that sharp R-line of the ^2^E → ^4^A_2_ emission band is rapidly quenched at around 50°C in contrary to the ^4^T_2_→^4^A_2_ emission. Therefore, due to this difference in the rates of thermal quenching of these particular emission bands, their luminescence intensity ratio was chosen as a temperature sensor LIR_1_:

(4) $$LIR_{1}=\frac{Cr^{3+}{(}^{4}T_{2}\to^{4}A_{2})}{Cr^{3+}{(}^{2}E\to^{4}A_{2})}=\frac{\int I(850-855)nm}{\int I(730-735)nm}$$

At low Cr^3+^ concentration the LIR_1_ decreases with temperature due to the fact that ^2^E state population feeds the ^4^T_2_ state at higher thermal energy. However, for higher Cr^3+^ (above 6%) different tendency can be found. Initially the LIR_1_ increases up to temperatures around 100°C above which saturation of its value can be found. This effect occurs because ^4^T_2_→^4^A_2_ band's intensity starts to play an important role in the total emission intensity. Due to the strong electron-phonon coupling, this emission band is expected to be efficiently reduced by the temperature. Therefore, its much higher rate of thermal quenching in respect to the ^2^E → ^4^A_2_ results in the enhancement of LIR_1_ value. To quantitative describe the observed changes, relative sensitivity (S) of LIR_1_-based luminescent thermometer was calculated according to the following formula:

(5) $$\begin{array}{r} S\left(T\right)=\frac{1}{LIR}\frac{\Delta LIR}{\Delta T}100\% \end{array}$$

Independently from the Cr^3+^ concentration, the relative intensities reach maximal value at temperatures below 100°C. Above this value, low changes of LIR_1_ are manifested as a minor value of S (Figure 3C). It is clearly seen that, according to our expectation, S significantly increases proportionally to Cr^3+^ content. The S_max_ at 9°C increases from 0.2%/°C for Y_3_Al_2_Ga_3_O_12_: 0.06%Cr^3+^ to 2.2%/°C for Y_3_Al_2_Ga_3_O_12_: 30%Cr^3+^ nanocrystals. Analogous tendency was found for the Y_3_Al_5_O_12_:Cr^3+^ and Y_3_Ga_5_O_12:_Cr^3+^ where for 10% Cr^3+^, S_max_ equals to 2.7%/°C at −105°C and 2%/°C at −78°C respectively (Figures S15, S16). The observed enhancement of the rate of the thermal quenching is obviously caused by the lowering of the CF strength. The optically active ions in the heavily Cr^3+^-doped nanocrystals are located in the lower CF sites (Figure 3D) which reduce the activation energy and facilitate luminescence thermal quenching. Therefore, the highest S were found for high Cr^3+^ concentration (low Dq/B values). However, above 130°C this correlation is suppressed due to the fact that above this temperature no ^2^E → ^4^A_2_ emission was observed. It is worth noting that the temperature range of high S overlaps with physiological temperature range (10–50°C) what indicates the importance of these LTs for biomedical applications.

**Figure 4:**
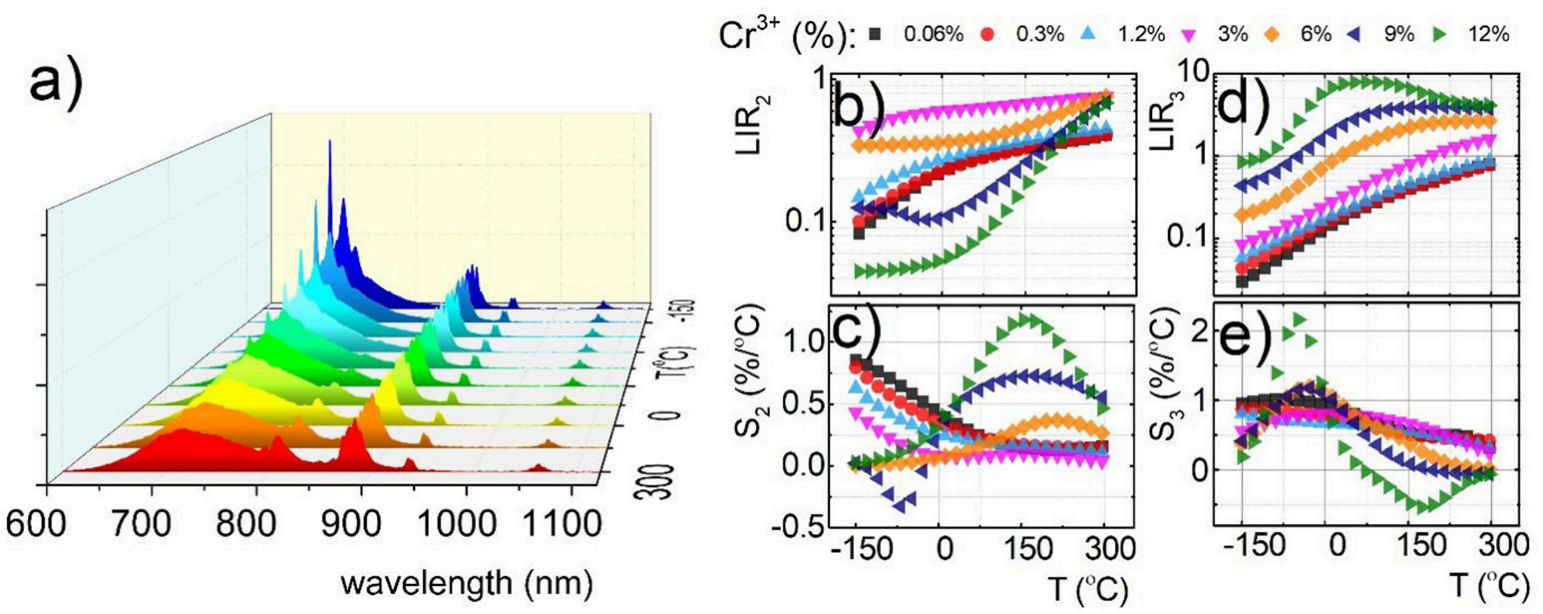
Comparison of thermal evolution of emission spectra for Y_3_Al_2_Ga_3_O_12_:1.2%Cr^3+^, 1%Nd^3+^ **(a)**; thermal evolution of LIR_2_ **(b)**; and LIR_3_ **(d)** and the corresponding relative sensitivities S_2_ **(c)** and S_3_ **(e)** for different Cr^3+^ concentration for Y_3_Al_2_Ga_3_O_12_:1.2%Cr^3+^, 1%Nd^3+^ nanocrystals.

Due to the fact that emission intensity of both ^2^E → ^4^A_2_ and ^4^T_2_→^4^A_2_ bands decreases at higher temperatures, relative sensitivity of luminescent thermometer based on their intensity ratio is reduced. Therefore, Nd^3+^ co-dopant, whose emission intensity is expected to be less temperature dependent, were used as a luminescent reference. The luminescent properties of Nd^3+^, Cr^3+^ co-doped nanocrystals were investigated in the analogous temperature range as in the case of singly Cr^3+^ doped counterparts. Representative thermal evolution of Y_3_Al_2_Ga_3_O_12_: 1.2%Cr^3+^, 1%Nd^3+^ nanocrystals is presented in Figure 4a. According to the expectations the intensity of bands at 880 and 1,060 nm attributed to ^4^F_3/2_→^4^I_9/2_ and ^4^F_3/2_→^4^I_11/2_ electronic transition of Nd^3+^ ions, respectively, is almost independent on the temperature, while the ^2^E → ^4^A_2_ emission intensity is strongly thermally quenched. It is worth noting that at temperatures above 50°C, additional Nd^3+^ bands appears which can be attributed to the ^4^F_5/2_, ^4^S_3/2_→^4^I_9/2_ electronic transition. This band occurs at higher temperature due to the fact that population of ^4^F_5/2_, ^4^S_3/2_ states increases in respect to the ^4^F_3/2_ with temperatures according to Boltzmann population. Taking advantage from these changes of the emission spectra with temperature, two types of luminescent thermometers based on the Nd^3+^/Cr^3+^ luminescence intensity ratio have been defined as follows:

(6) $$LIR_{2}=\frac{Nd^{3+}{(}^{4}F_{3/2}\to^{4}I_{9/2})}{Cr^{3+}{(}^{4}T_{2}\to^{4}A_{2})}=\frac{\int I(869-869.5)nm}{\int I(705-705.5)nm}$$

(7) $$LIR_{3}=\frac{Nd^{3+}{(}^{4}F_{5/2}\to^{4}I_{9/2})}{Cr^{3+}{(}^{4}T_{2}\to^{4}A_{2})}=\frac{\int I(810-810.5)nm}{\int I(710-710.5)nm}$$

The thermal dependence of LIR_2_ and LIR_3_ for different concentration of Cr^3+^ ions are presented in Figures 4b,d, respectively. Obviously, due to the Cr^3+^ → Nd^3+^ energy transfer the usable concentration range of Cr^3+^ is strongly limited (to 12% Cr^3+^). Independently from dopant concentration, both LIR_2_ and LIR_3_ increase at higher temperature due to the thermal quenching of ^4^T_2_→^4^A_2_ band of Cr^3+^ (Figures 4b,d). However, more rapid changes can be found for LIR_3_ what is obviously related with the increase of the ^4^F_5/2_, ^4^S_3/2_→^4^I_9/2_ emission band of Nd^3+^. Both LIR_2_ and LIR_3_ are strongly modulated by the Cr^3+^ concentration. In agreement with the results obtained for singly Cr^3+^ doped systems, significant enhancement of LIR's thermal changes can be found. At heavily doped phosphors the activation energies of ^4^T_2_ level is meaningfully diminished facilitating its nonradiative depopulation. Small activation energy is beneficial for enhancement of the relative sensitivity of luminescent thermometers. Therefore, these effects substantially affect the relative sensitivity of both S_2_ and S_3_ (Figures 4c,e). The highest S_2_ at 150°C increases from 0.17%/°C for 0.06% of Cr^3+^ to 1.17%/°C for 12% of Cr^3+^ ions. On the other hand the S_3_ at −48°C increases from 0.95%/°C for 0.06% of Cr^3+^ to 2.16%/°C for 12% of Cr^3+^ ions. Comparing these results with the Figure 3, evident profitable effect of the Nd^3+^ ions use as a luminescent reference can be found. The relative sensitivity for 0.06% Cr^3+^ increases from *S*_1_ = 0.14%/°C to *S*_2_ = 0.17%/°C and *S*_3_ = 0.95%/°C, while for 12% Cr^3+^ from *S*_1_ = 1.37%/°C to *S*_2_ = 1.17%/°C and *S*_3_ = 2.16%/°C. Analogous beneficial effect of high Cr^3+^ concentration can be found for Y_3_Ga_5_O_12:_Cr^3+^, Nd^3+^ as well as Y_3_Al_5_O_12:_Cr^3+^, Nd^3+^ (Figures S10, S11). It is also worth noting that the LIR_2_ reveals high sensitivity at high temperature range (above 100°C) in contrast to LIR_3_, which unveils good performance for non-contact temperature sensing at low temperatures (below 0°C). Therefore, by simultaneous employment of both of these thermometers the temperature range where high accuracy temperature readout can be achieved, is widened.

The highest recorded sensitivity (*S* = 2.64%/°C) was found for YAG nanocrystals at −100°C for 10% of Cr^3+^ (Figure S15B) due to the fact of the lowest CF strength for this host material. Nevertheless, CF strength was tuned in the widest range for Y_3_Al_2_Ga_3_O_12_: Cr^3+^, Nd^3+^ via the Cr^3+^ doping the enhancement of S_3_ was the strongest in this case. The most important finding presented in this paper is that highly sensitive luminescent thermometers can be intentionally designed by the modification of the CF strength through elongation of Cr^3+^-O^2−^ distance and enlargement of the Cr^3+^ concentration.

## Conclusions

In this work, we proposed a new strategy to modulate the relative thermal sensitivity of Cr^3+^ doped nanophosphors. We considered three types of garnet matrices doped with different Cr^3+^ concentrations, with or without Nd^3+^ co-dopant. It was shown that the increase of the Cr^3+^ concentration causes elongation of the average Cr^3+^-O^2−^ distance leading to the reduction of the crystal field strength in Y_3_Al_2_Ga_3_O_12_, Y_3_Ga_5_O_12_ and Y_3_Al_5_O_12_ nanocrystals. A gradual increase of the broad emission band attributed to the ^4^T_2_→^4^A_2_ spin allowed transition of Cr^3+^ is observed. Moreover, the reduction of the ^4^T_2_ state energy facilitates thermal quenching of ^2^E state. Therefore, the relative sensitivity of ^4^T_2_→^4^A_2_ to ^2^E → ^4^A_2_ emission intensity increases from *S* = 0.2%/°C for 0.06%Cr^3+^ to *S* = 2.2%/°C for 30%Cr^3+^ at 9°C in Y_3_Al_2_Ga_3_O_12_ nanocrystals, from *S* = 0.027%/°C for 0.5%Cr^3+^ to *S* = 2.7% for 10%Cr^3+^ at −105°C in Y_3_Al_5_O_12_ nanocrystals, and from *S* = 0.14%/°C for 0.5%Cr^3+^ to *S* = 2% for 10%Cr^3+^ at −78°C in Y_3_Ga_5_O_12_ nanocrystals. In the case of Nd^3+^ co-doped nanocrystals, due to the Cr^3+^ → Nd^3+^ energy transfer, the usable concentration range of Cr^3+^ dopants is strongly reduced and no evidence of Cr^3+^ emission was found above 10% of Cr^3+^. However, by taking advantage from this energy transfer and the fact that ^4^F_5/2_, ^4^S_3/2_→^4^I_9/2_ emission intensity increases proportionally to the temperature according to the Boltzmann distribution, the relative sensitivities of luminescent thermometers defined as ^4^F_5/2_, ^4^S_3/2_→^4^I_9/2_ to ^4^T_2_→^4^A_2_ luminescence intensity ratio enhances from 1.3%/°C at −50°C without Nd^3+^ dopant to 2.2%/°C at −50°C for nanocrystals doped with Nd^3+^ ions Y_3_Al_2_Ga_3_O_12_: 10%Cr^3+^, Nd^3+^). The presented results confirm that the relative sensitivity of luminescent thermometers can be effectively modulated by the dopant concentration. Moreover, it was proved that the presence of Nd^3+^ dopant contributes to faster quenching of Cr^3+^ luminescence, what is favorable for highly sensitive non-contact luminescent thermometers. Our studies may be considered as a next step toward intentional designing of nanocrystalline luminescent thermometers with fully controllable thermooptical response.

## Author contributions

All authors listed have made a substantial, direct and intellectual contribution to the work, and approved it for publication.

### Conflict of interest statement

The authors declare that the research was conducted in the absence of any commercial or financial relationships that could be construed as a potential conflict of interest.
