# Supplementary material for: The Impact of Cr3+ Doping on Temperature Sensitivity Modulation in Cr3+ Doped and Cr3+, Nd3+ Co-doped Y3Al5O12, Y3Al2Ga3O12, and Y3Ga5O12 Nanothermometers
Source: Front Chem. 2018 Sep 19;6:424. doi: 10.3389/fchem.2018.00424 (PMC6157326; doi:10.3389/fchem.2018.00424)
Supplement: Supplementary file 1 [file Table_1.DOCX]

**SUPPORTING INFORMATION**

**The impact of Cr^3+^ doping on temperature sensitivity modulation in Cr^3+^ doped and Cr^3+^,Nd^3+^ co-doped Y_3_Al_5_O_12_, Y_3_Al_2_Ga_3_O_12_ and Y_3_Ga_5_O_12_ nanothermometers**

**K. Elzbieciak^1^, L. Marciniak^1*^**

*^1^ Institute of Low Temperatures and Structure Research PAS*

***e-mail** [**l.marciniak@int.pan.wroc.pl**](mailto:l.marciniak@int.pan.wroc.pl)

**
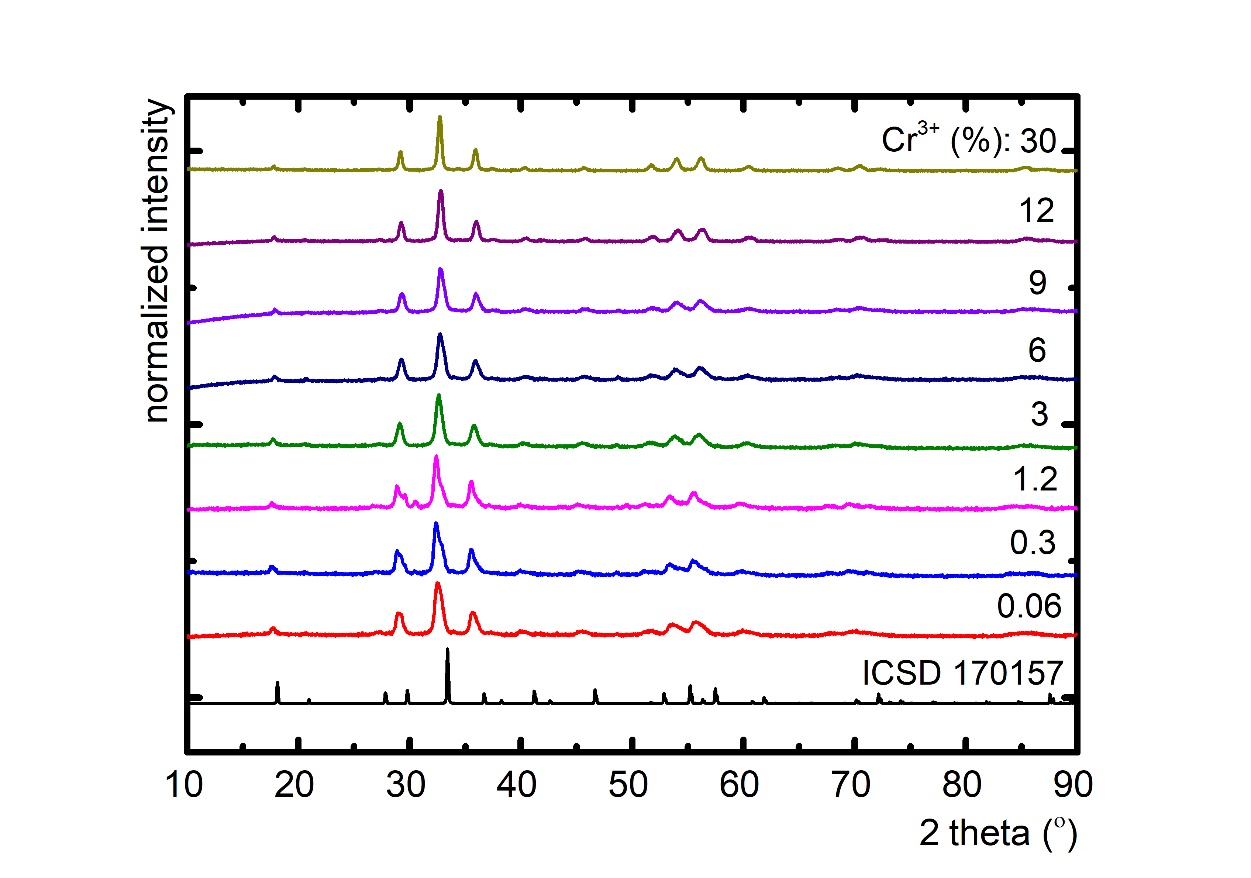
**

**Figure S.1.** XRD patterns of Y_3_Al_2_Ga_3_O_12_: Cr^3+^ with different Cr^3+^ concentration.


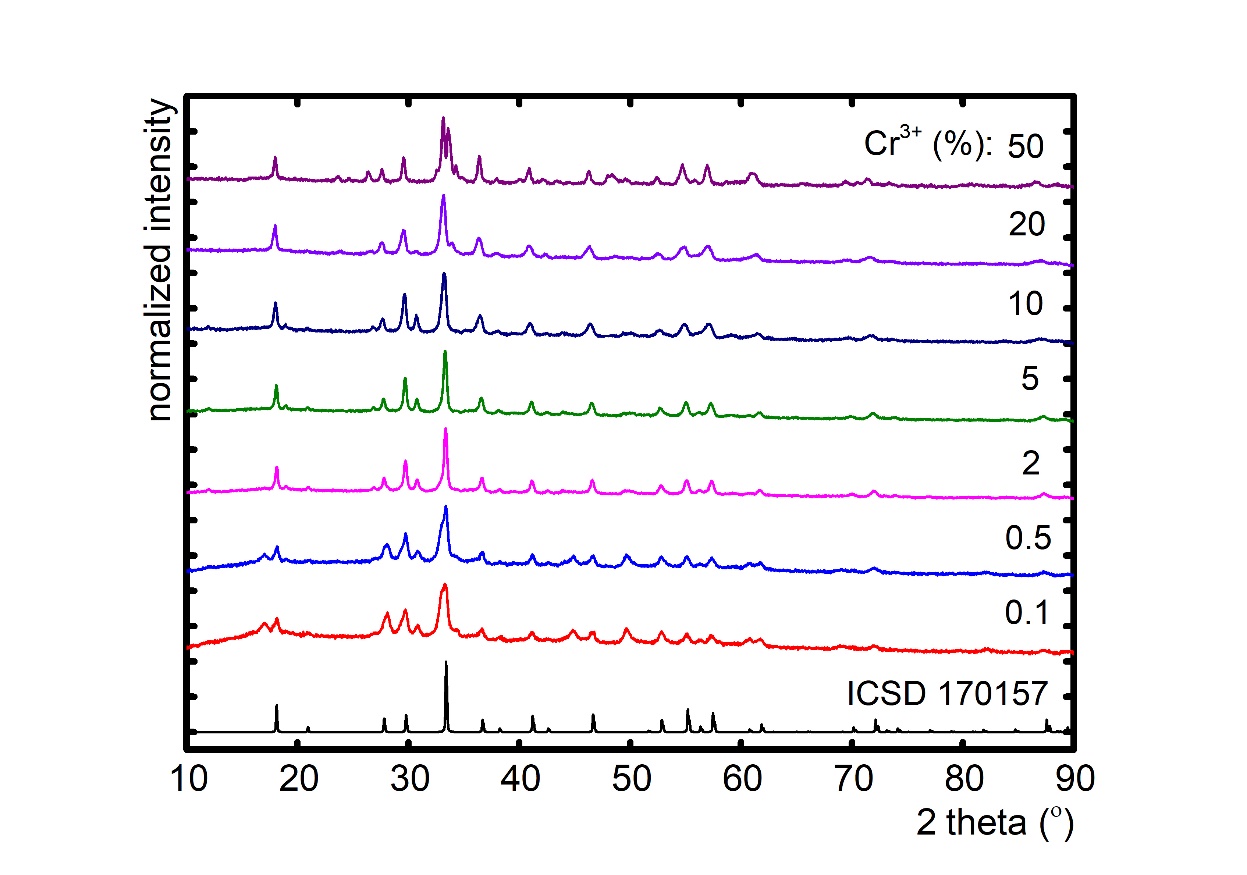


**Figure S.2.** XRD patterns of Y_3_Al_5_O_12_: Cr^3+^ with different Cr^3+^concentration.


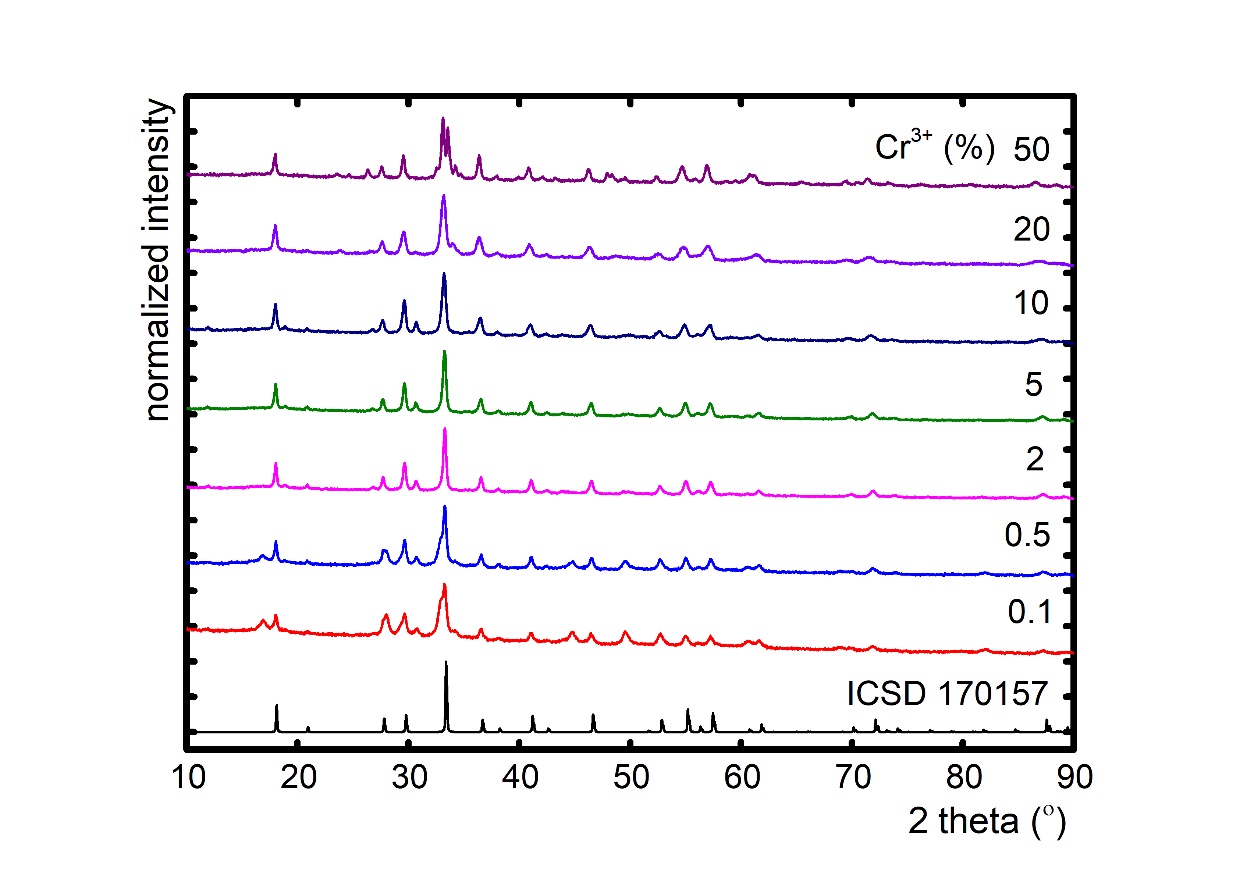


**Figure S.3.** XRD patterns of Y_3_Al_5_O_12_: Cr^3+^, 1%Nd^3+^ with different Cr^3+^ concentration.


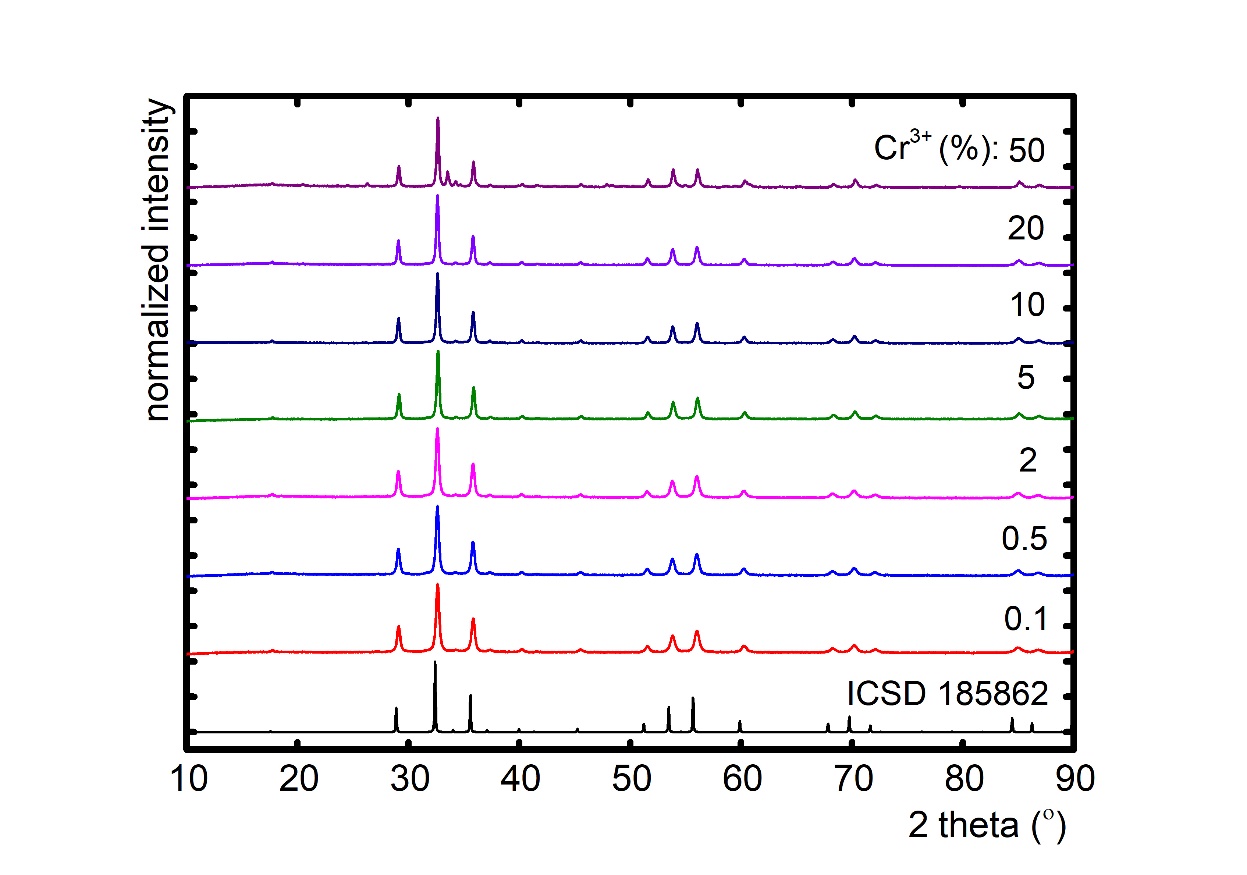


**Figure S.4.** XRD patterns of Y_3_Ga_5_O_12_: Cr^3+^ with different Cr^3+^ concentrations.


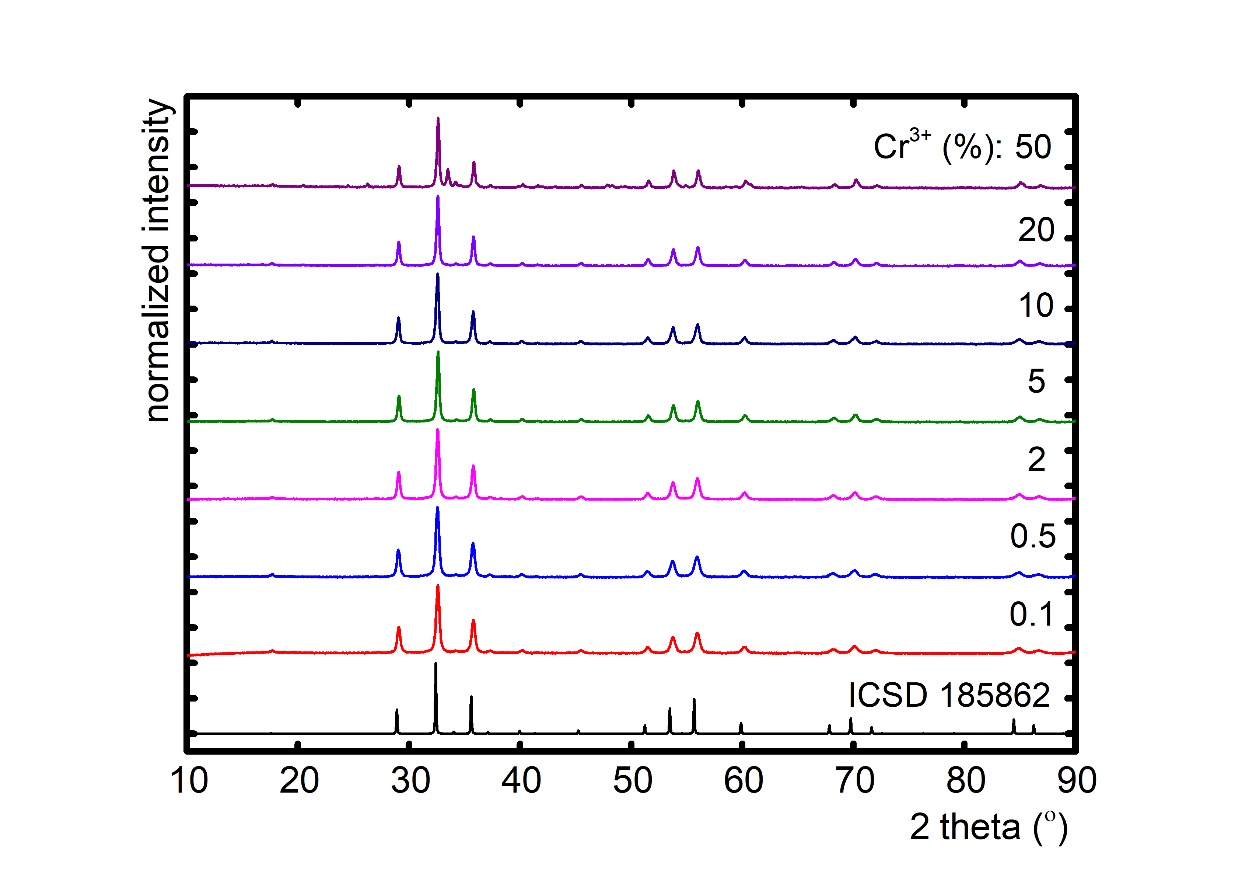


**Figure S.5.** XRD patterns of Y_3_Ga_5_O_12_: Cr^3+^, 1%Nd^3+^ with different Cr^3+^ concentrations.


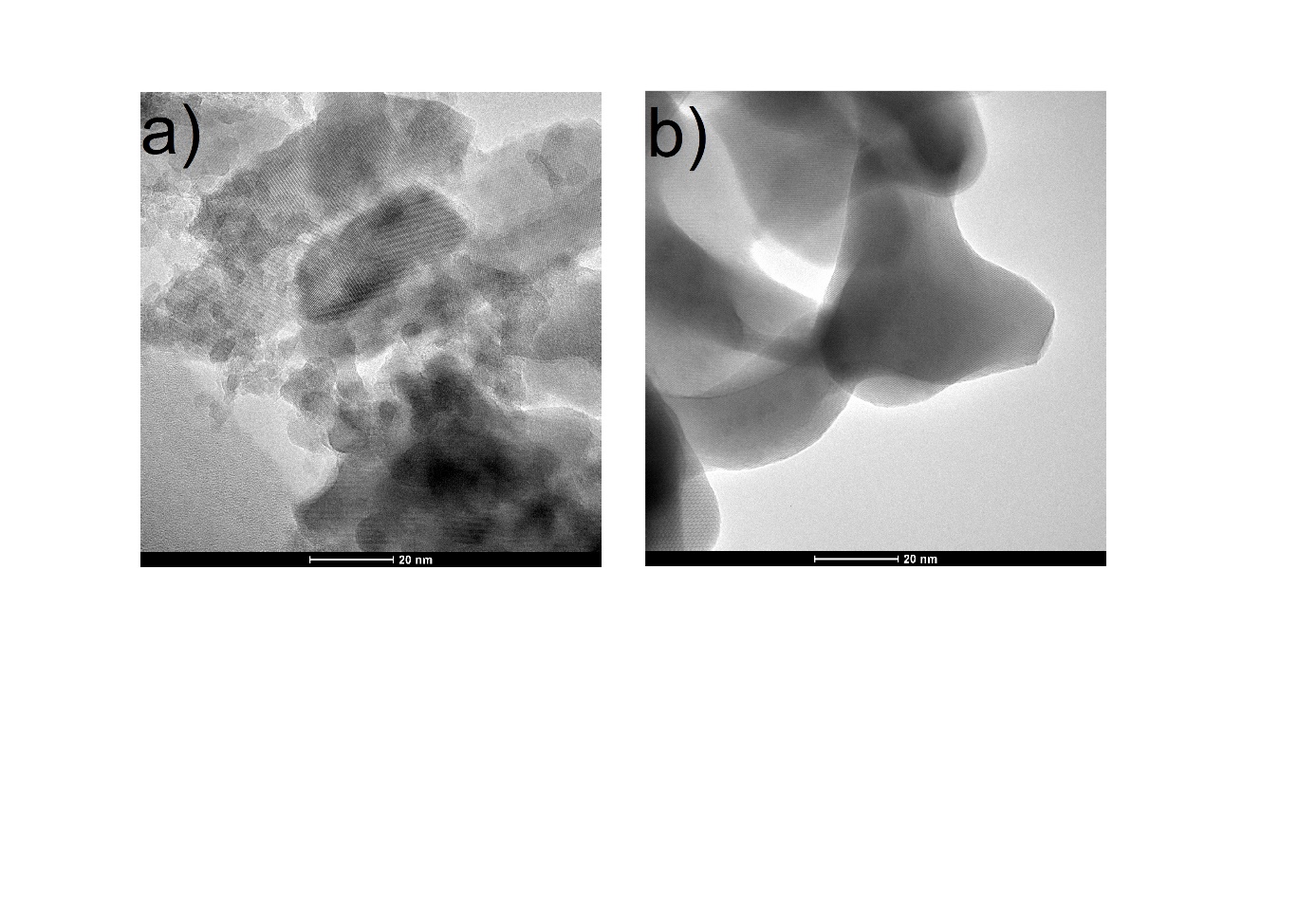


**Figure S.6.** Representative TEM images of YAG:Cr^3+^-a) and YGG:Cr^3+^-b) nanocrystals.


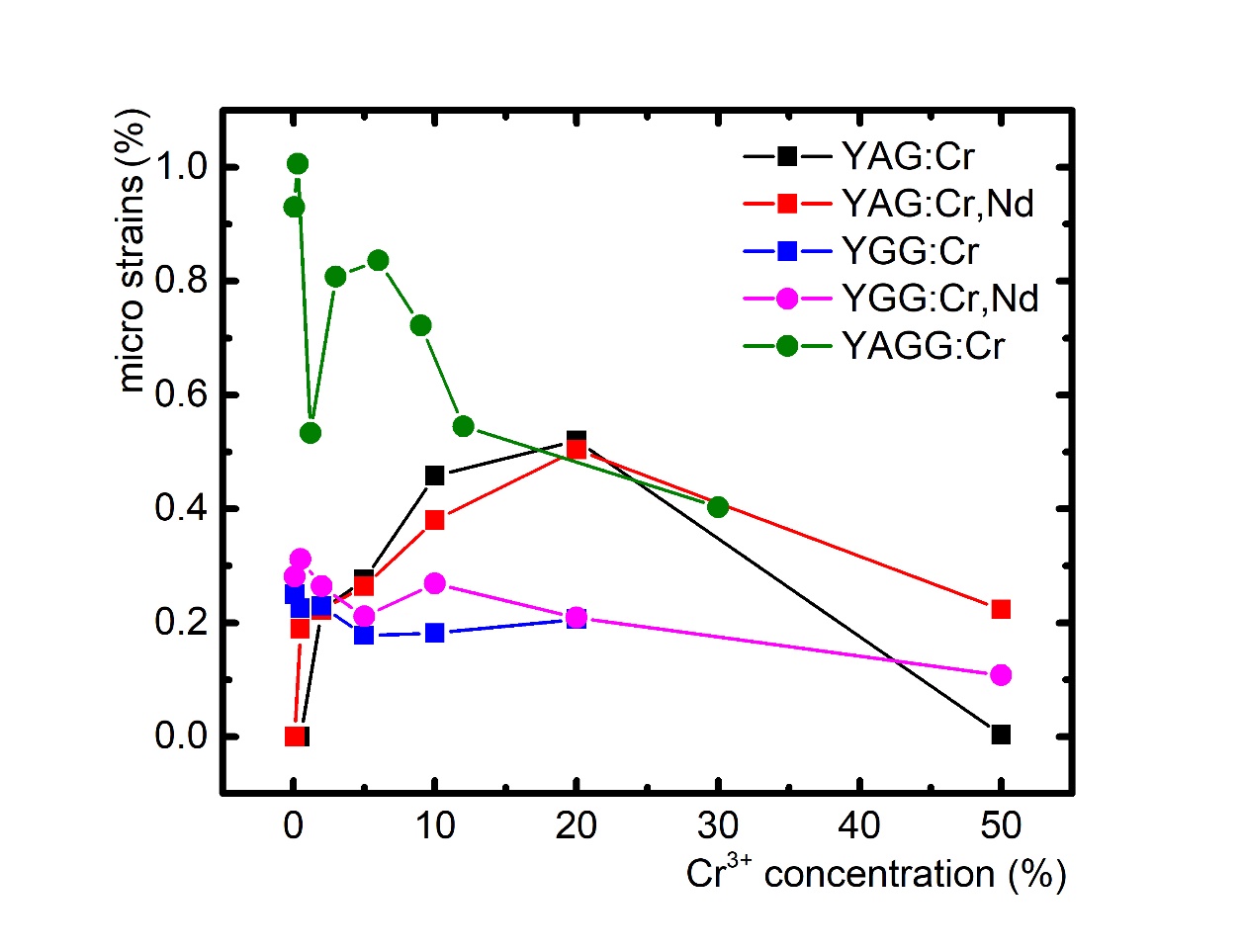


**Figure S.7.** The dependence of micro strains on Cr^3+^ concentration for different host materials.


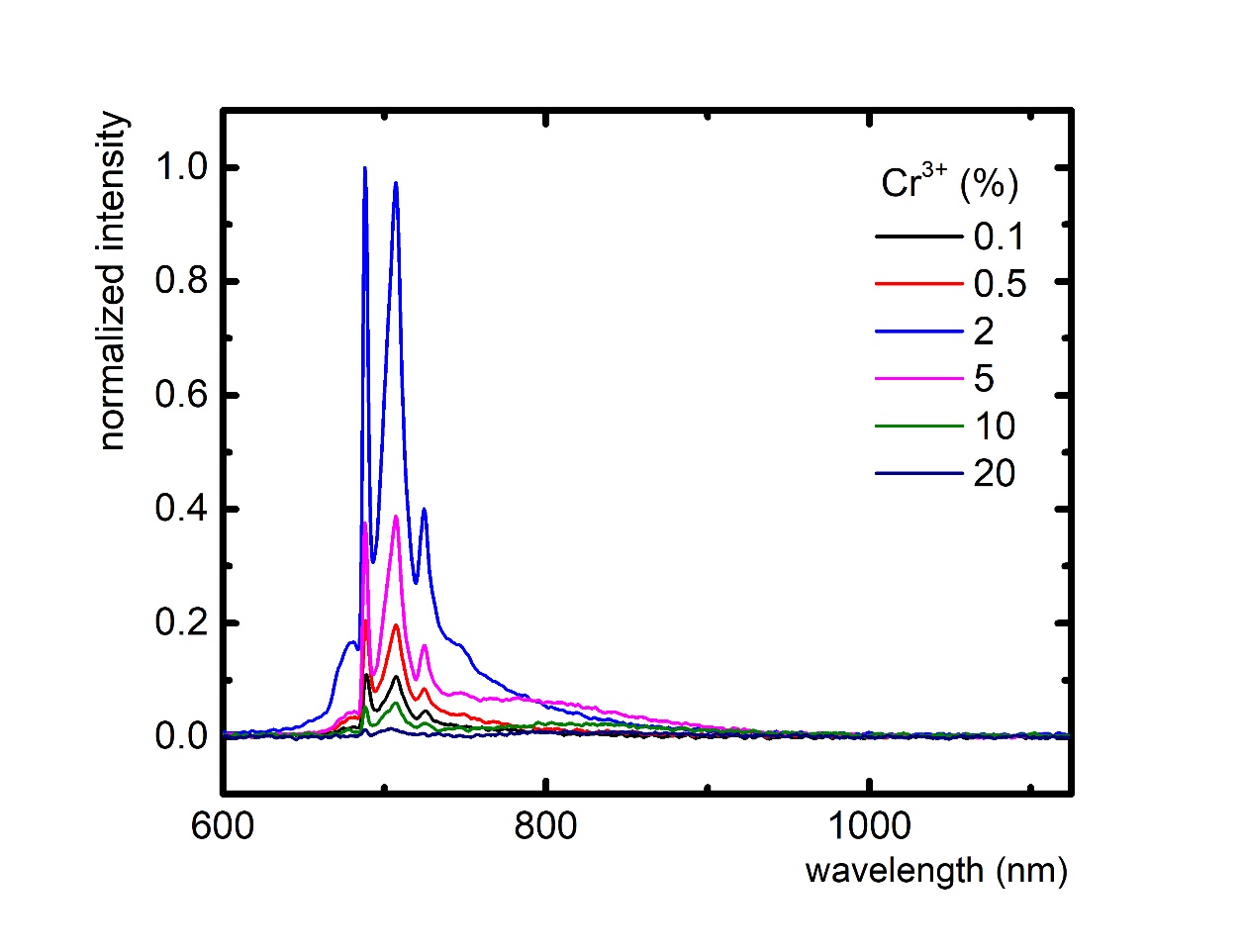


**Figure S.8.** Comparison of Y_3_Al_5_O_12_: Cr^3+^ emission spectra with different concentration of Cr^3+^ measured at -150 ^o^C.


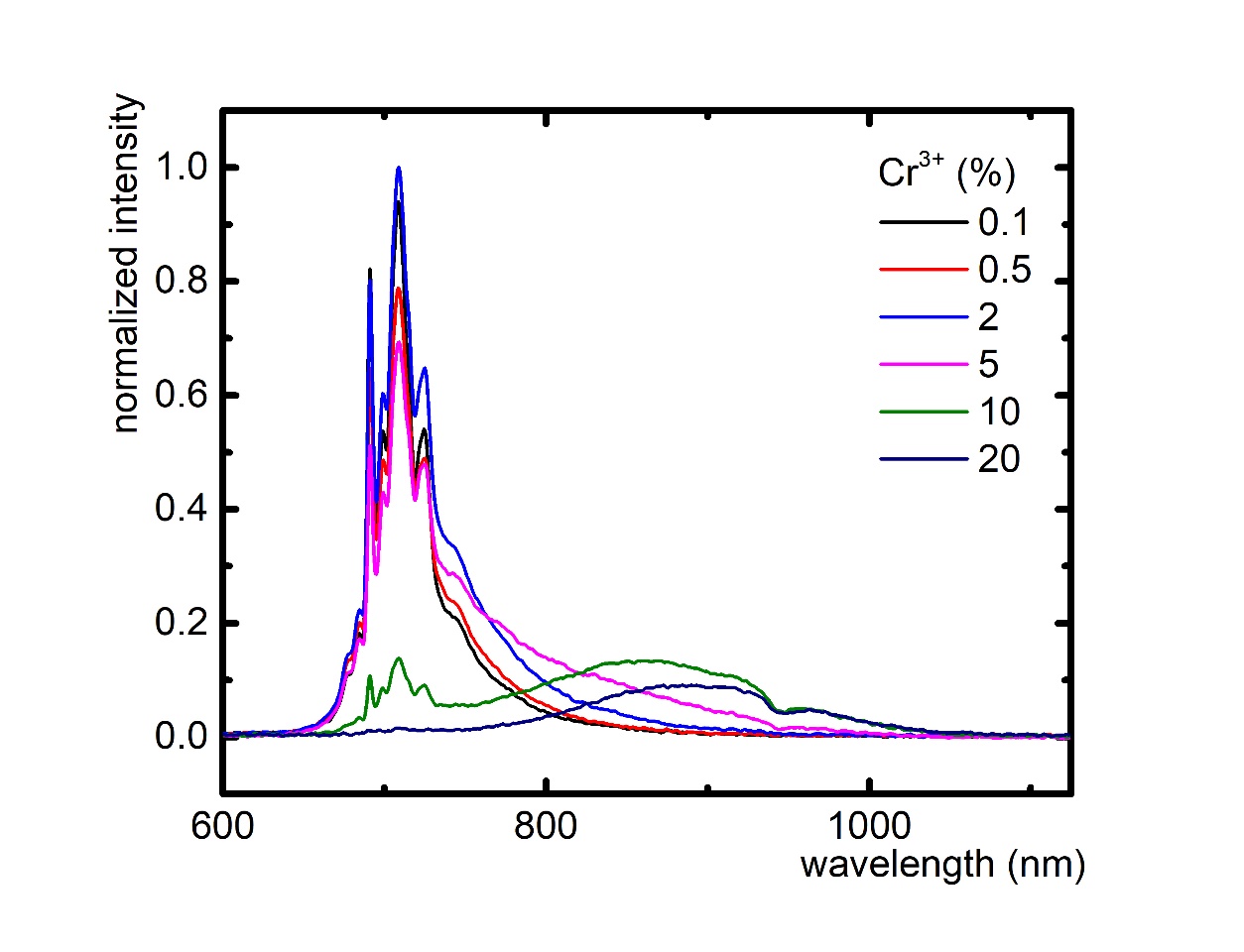


**Figure S.9.** Comparison of Y_3_Ga_5_O_12_: Cr^3+^ emission spectra with different concentration of Cr^3+^ dopant, measured at -150 ^o^C.


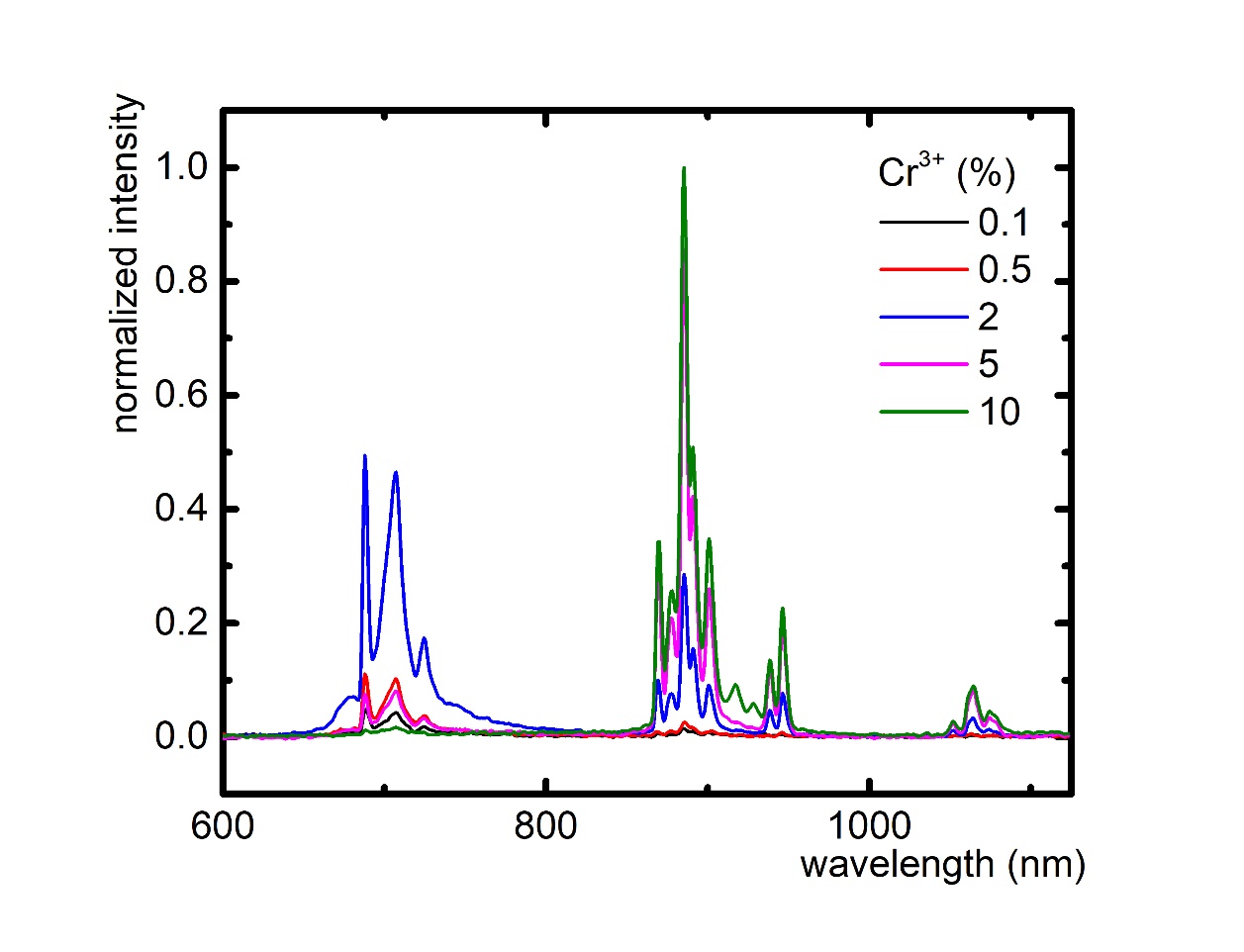


**Figure S.10.** Comparison of Y_3_Al_5_O_12_: Cr^3+^, 1%Nd^3+^ emission spectra with different concentration of Cr^3+^ measured at -150 ^o^C.


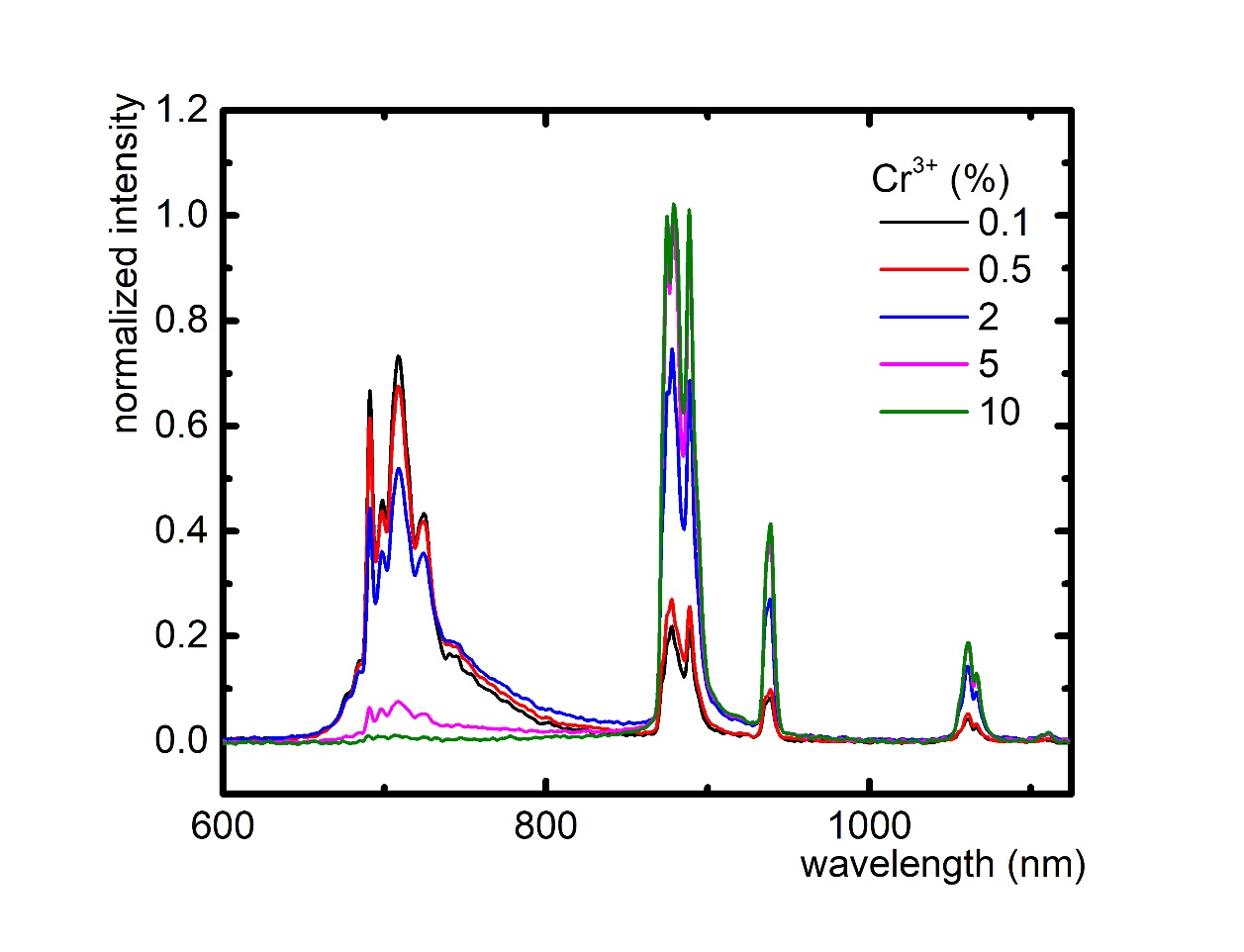


**Figure S.11.** Comparison of Y_3_Ga_5_O_12_: Cr^3+^, 1%Nd^3+^ emission spectra with different concentration of Cr^3+^ measured at -150 ^o^C.


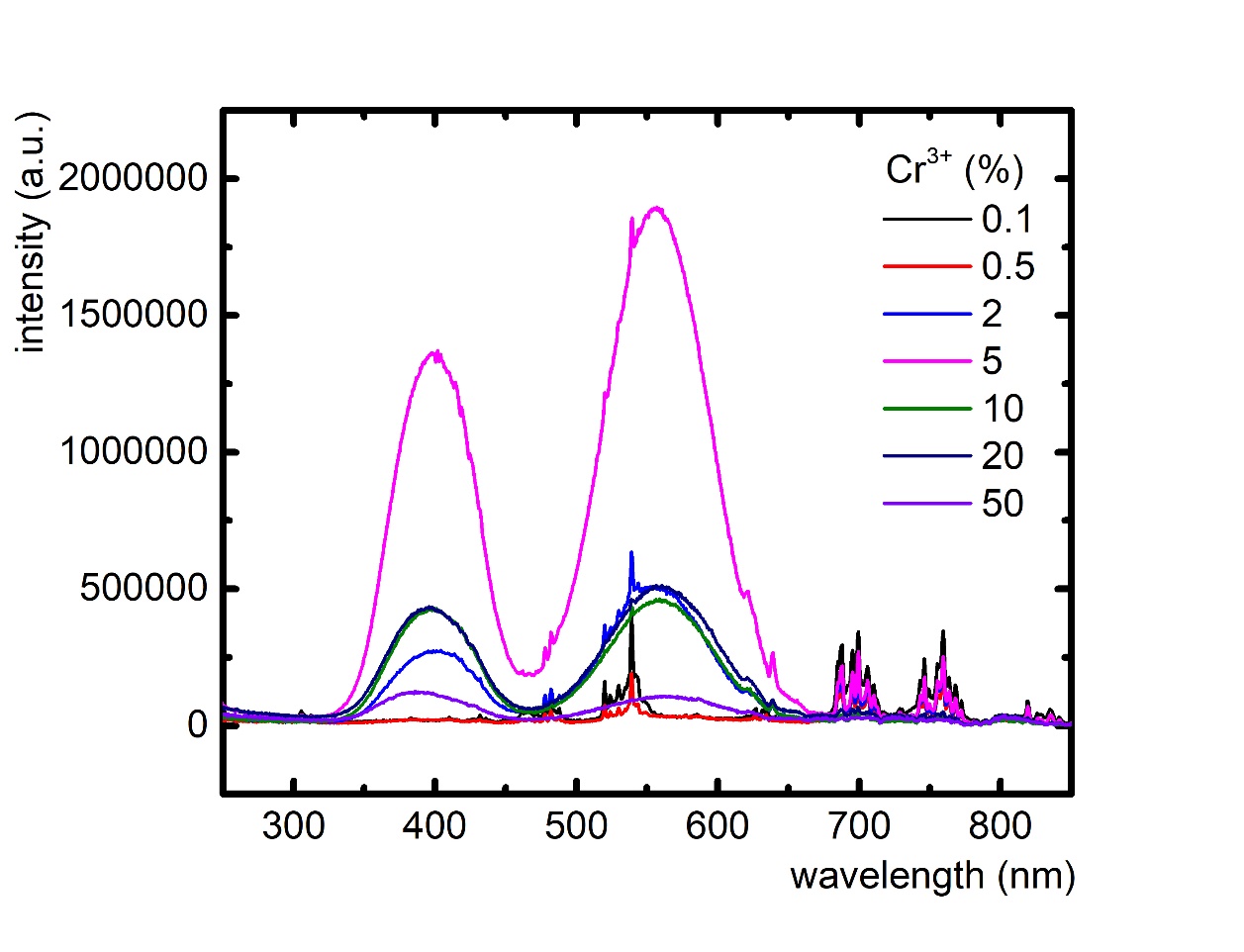


**Figure S.12.** Comparison of normalized excitation spectra of Y_3_Al_5_O_12_: Cr^3+^, 1%Nd^3+^ with different concentration of Cr^3+^, detected at 1064 nm.


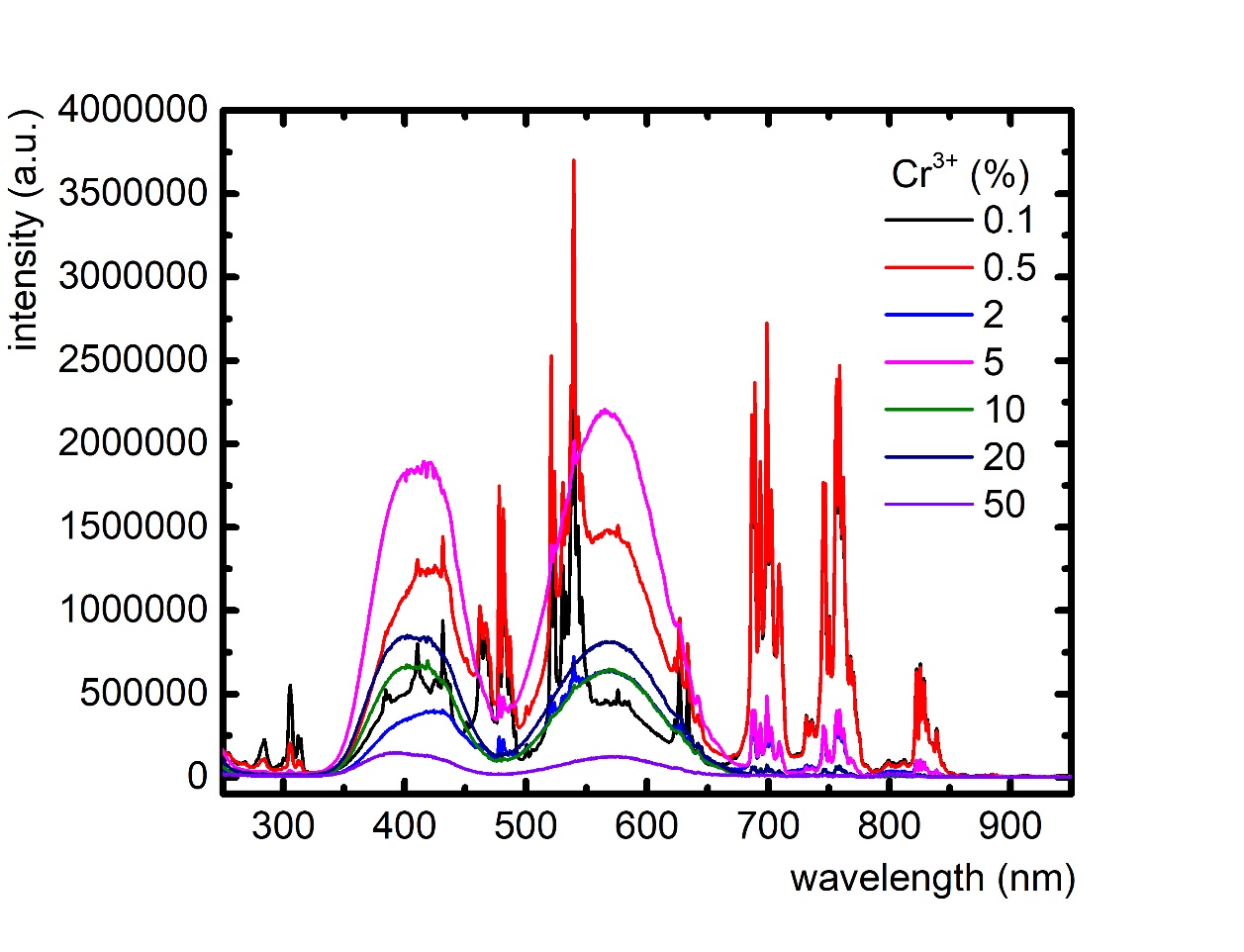


**Figure S.13.** Comparison of normalized excitation spectra of Y_3_Ga_5_O_12_: Cr^3+^, 1%Nd^3+^ with different concentration of Cr^3+^, detected at 1064 nm.


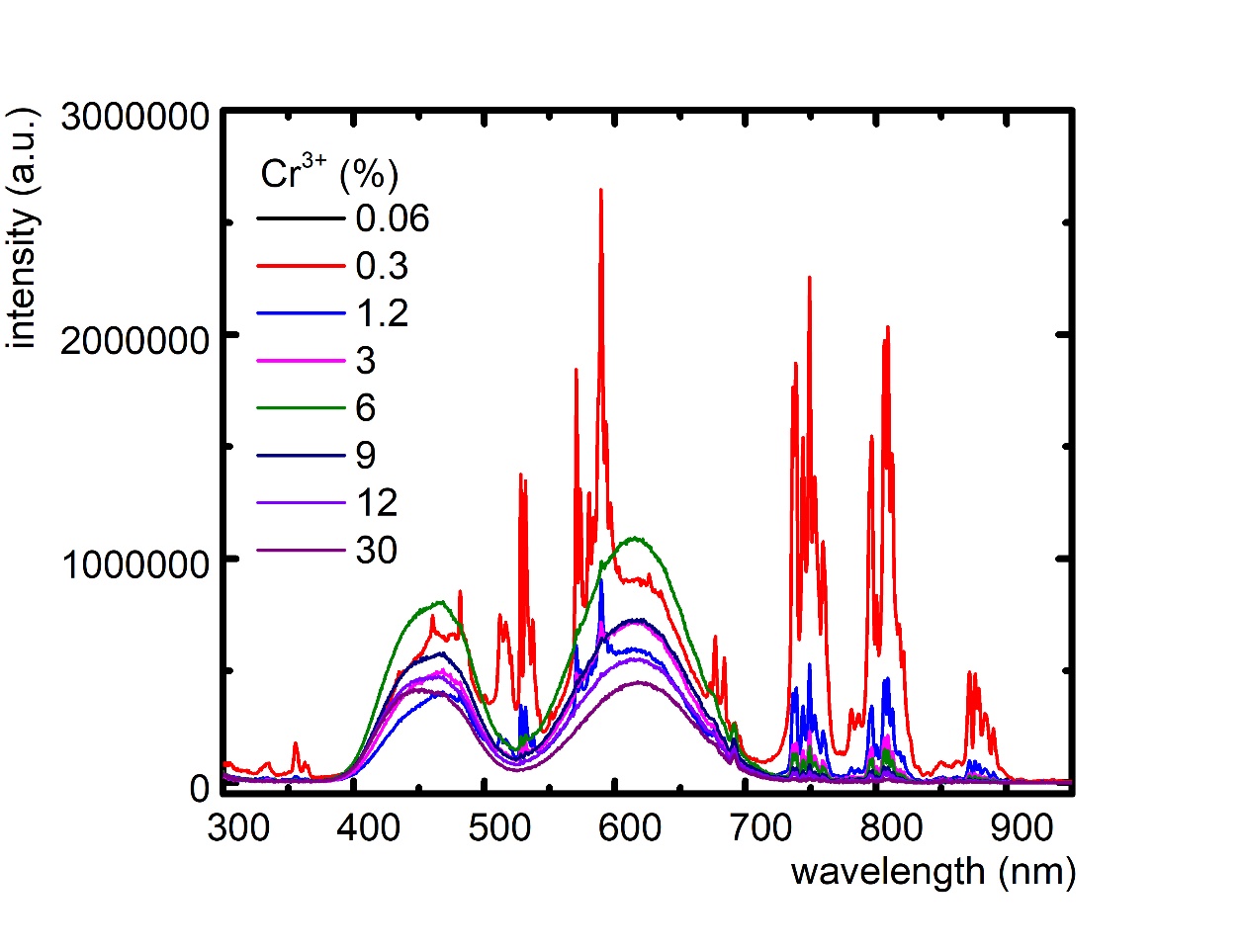


**Figure S.14.** Comparison of normalized excitation spectra of Y_3_Al_2_Ga_3_O_12_: Cr^3+^, 1%Nd^3+^ with different concentration of Cr^3+^, detected at 1064 nm.


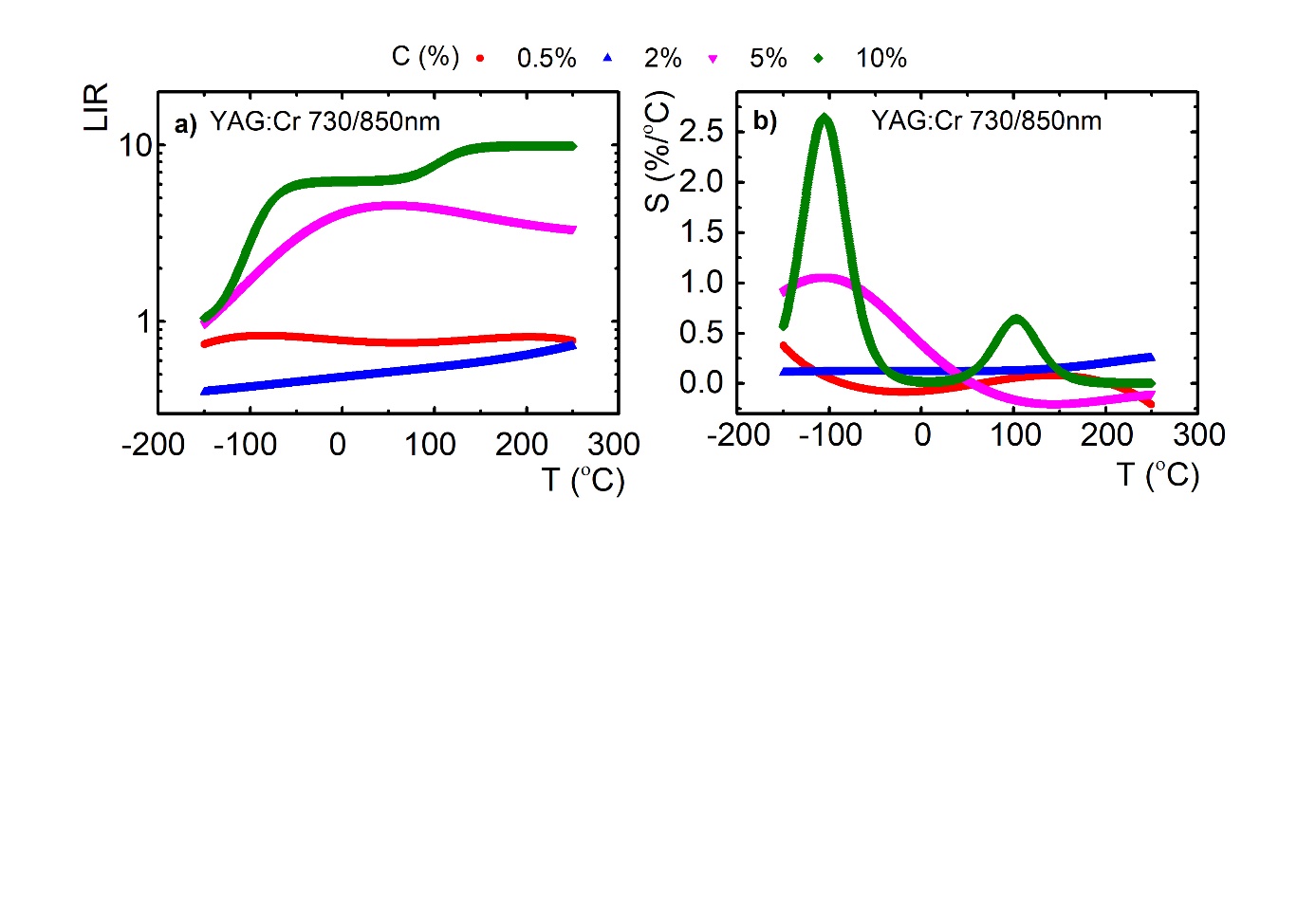


**Figure S.15.** Thermal evolution of LIR -a) with the corresponding relative sensitivities –b).


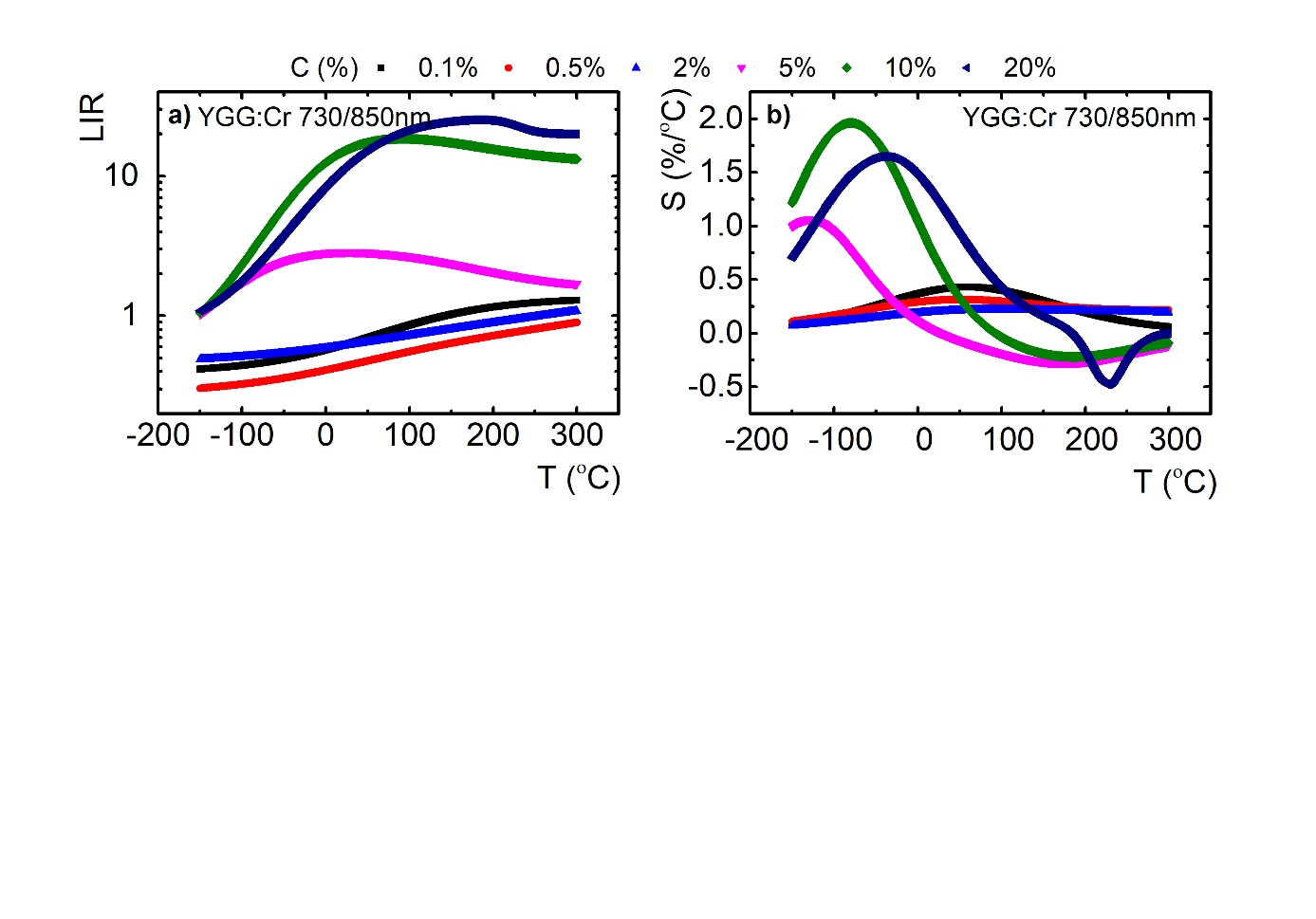


**Figure S.16.** Thermal evolution of LIR -a) with the corresponding relative sensitivities –b).
